# Lateral versus posterior quadratus lumborum block in children undergoing open orchiopexy: a double-blind randomized clinical trial

**DOI:** 10.1016/j.bjane.2025.844661

**Published:** 2025-07-05

**Authors:** Ozgecan P. Zanbak Mutlu, Pinar Kendigelen, Ayse C. Tutuncu

**Affiliations:** Istanbul University-Cerrahpasa, Cerrahpasa Faculty of Medicine, Department of Anesthesiology and Reanimation, Istanbul, Turkey

**Keywords:** Acute pain, Analgesia, Nerve block, Orchiopexy, Pediatrics

## Abstract

**Background:**

Quadratus Lumborum Block (QLB) has recently become an effective analgesic regional technique frequently used in abdominal surgeries. However, due to the heterogeneity in studies regarding block approaches, a direct comparison of QLB types is needed. In this double-blind prospective randomized trial, we aimed to compare the effects of lateral and posterior approaches of QLB on pain and analgesic use in children undergoing orchiopexy.

**Methods:**

Patients aged 6 months – 12 years undergoing elective unilateral open orchiopexy were included in the study. Patients were randomized into two groups using the closed-envelope method. Lateral or posterior QLB was applied under ultrasonography with 0.4 mL/kg 0.25% bupivacaine for both groups before the surgery. The primary outcome was the assessment of postoperative pain for 24 hours. Analgesic usage, parental satisfaction, and complications were recorded as secondary outcomes.

**Results:**

Analyses were conducted on 80 patients. Both study groups achieved clinically adequate analgesia, and no significant pain score distinctions were observed within 24 hours (Total mean scores: FLACC [lateral QLB: 2.86 ± 4.69 vs. posterior QLB: 2.87 ± 3.71, p = 0.466], Wong-Baker [lateral QLB: 0.86 ± 2.03 vs. posterior QLB: 1.24 ± 1.85, p = 0.151]). No significant interaction effect between groups and postoperative time intervals on pain scores was observed (FLACC score p-interaction: 0.425, Wong-Baker score p-interaction: 0.451). There were no statistical differences in the number of patients necessitating intraoperative and postoperative analgesics. Parental satisfaction exhibited similarity between the groups, and no perioperative complications were observed in either group.

**Conclusion:**

Lateral and posterior QLB provided similar perioperative analgesia in pediatric patients undergoing orchiopexy.

**Clinical trial registration number:**

NCT05056038.

**Date of registration:**

02 June 2021.

## Introduction

Pediatric patients exhibit a heightened response to pain stimulation, and potential barriers exist in managing pain, often resulting in undertreatment.^1^ Postoperative pain is associated with complications, delayed recovery, diminished patient satisfaction, and chronic pain.^2^ Hence, prioritizing effective pain management is crucial in children. Orchiopexy, a commonly performed surgical procedure, targets an anatomical region characterized by extensive and complex innervation, posing challenges for pain control.^3^^,^^4^

Quadratus Lumborum Block (QLB) is a recently described fascial plane block that has been shown to be an effective and reliable analgesic method for lower abdominal surgeries and orchiopexy in pediatric patients.^2^^,^^4^^,^^5^ Numerous studies have demonstrated that QLB is more effective and longer-lasting compared to the caudal block.^6^

The concept of QLB was initially introduced by Blanco, and since then, different variations of QLB have been defined by administering injections on various sides of the Quadratus Lumborum Muscle (QLM).^7^^,^^8^ The mechanisms and analgesic efficacy of various QLB approaches remain controversial in the current literature.^2^^,^^7^, ^8^, ^9^, ^10^ As of now, no conclusive evidence supporting one QLB type over the other.^9^

The aim of the study was to assess and compare the analgesic effectiveness of ultrasound-guided lateral and posterior QLB approaches in pediatric patients undergoing orchiopexy. We hypothesized that posterior QLB could provide better analgesia with a more extensive spread compared to lateral QLB.^7^^,^^11^^,^^12^

## Methods

This prospective study, designed as a randomized, double-blind trial, was approved by the Istanbul University-Cerrahpasa, Institutional Review Board (IRB #90211). Written informed consent was obtained from the parents or legal guardians of all patients who participated in the trial. The study was registered on clinicaltrials.gov (NCT05056038, date of registration: June 2021) before enrolling patients, and the manuscript adheres to the CONSORT guidelines, employing a flow diagram for patient enrolment and allocation.

The study included pediatric patients with the American Society of Anesthesiologists (ASA) class I‒III, aged between 6 months and 12 years, undergoing elective unilateral orchiopexy between July 2021 and July 2022. Exclusion criteria encompassed patients with contraindications for regional anesthesia, declined to provide consent, scheduled for a laparoscopic approach, ASA class IV, and requiring postoperative admission to the intensive care unit. The primary outcome was the assessment of postoperative pain for 24 hours. Analgesic usage, parental satisfaction, and complications were the secondary outcomes.

Patients were premedicated with intravenous 0.05 mg/kg midazolam and 0.5 mg/kg ketamine. Following standard monitorization, induction of anesthesia was achieved using 5 mg/kg thiopental, 1 μg/kg fentanyl, 0.6 mg/kg rocuronium, and subsequent orotracheal intubation was performed. Anesthesia was maintained with 2% sevoflurane. The duration of surgical procedures was recorded.

All blocks were performed by two highly experienced pediatric anesthesiologists (A.C.T and P.K) after endotracheal intubation and prior to the surgical procedure. The specific type of block ‒ either lateral or posterior QLB ‒ was determined using a sealed envelope technique. Each patient was assigned a study number to ensure anonymized tracking. Perioperative follow-up and data collection were conducted by a third anesthesiologist, who was blinded to group allocation, along with nursing staff. The anesthesiologists performing the blocks were aware of the group assignments solely to perform the correct intervention; however, they were not involved in any aspect of data collection. Furthermore, both patients and their parents remained blinded to group allocation throughout the study period.

Both techniques were performed in a supine or semi-lateral position under sterile conditions, utilizing 18, 20, or 22-gauge intravenous cannulas (Bicakcilar Cooperation, Istanbul, Turkey) selected based on the patient's age. The needle was guided using the linear probe of the ultrasound system (GE Logiq-E Ultrasound System with 9L Linear Transducer, Illinois, USA), and the 'in-plane' technique was employed. After the probe was positioned at the umbilical level, advanced until the terminal of the Transversus Abdominis Muscle (TAM) and QLM were visualized. The needle was directed anteroposteriorly. Following the confirmation of correct needle placement, ascertained by the absence of blood aspiration and injecting small aliquots of 1 mL 0.9% saline, both blocks were initiated with the administration of 0.4 mL/kg of 0.25% bupivacaine.

**Figure 1:**
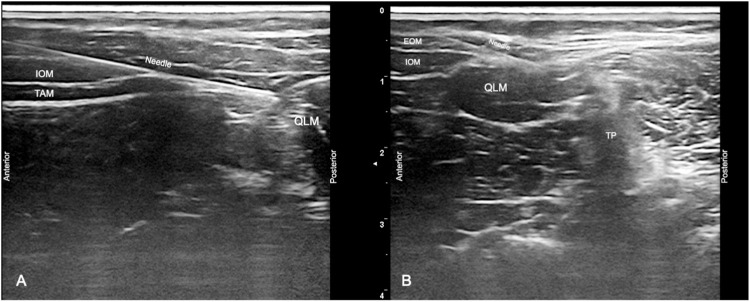
(A) USG imaging of the lateral QLB, (B) USG imaging of the posterior QLB. EOM, External Oblique Muscle; IOM, Internal Oblique Muscle; QLM, Quadratus Lumborum Muscle; TAM, Transversus Abdominis Muscle; TP, Transverse Process; USG, Ultrasonography.

Lateral QLB (QLB-1): Local Anesthetic (LA) is injected into the anterolateral aspect of the QLM, specifically at the junction with the posterior aponeurosis of the TAM and the transversalis fascia. The transversalis fascia merges with the QLM fascia to form the anterior Thoracolumbar Fascia (TLF) (Fig. 1A).^7^^,^^8^^,^^13^

Posterior QLB (QLB-2): LA is injected on the posterior surface of the QLM. This injection site is located between the QLM, erector spinae, and latissimus dorsi muscles, targeting a specific anatomical region called the Lateral Interfascial Triangle (LIFT). The LIFT represents a triangular structure located at the juncture of the middle TLF and the deep lamina of the posterior TLF (paraspinal retinacular sheath) (Fig. 1B).^7^^,^^8^^,^^13^

Following the administration of the block, the Mean Arterial Pressure (MAP) and Heart Rate (HR) were recorded before the surgical incision and at the 5, 10, 20, 30, 45, and 60 minutes after the incision. The surgery started at least 10 minutes after the performance of the block. In the event of a 20% increase in HR and MAP from the baseline, remifentanil infusion was initiated in accordance with current guideline recommendations.^14^ The dose of remifentanil was adjusted based on HR and MAP measurements (within ± 20% of the baseline), and the infusion was terminated as soon as possible.

Following surgery, all patients received standard postoperative care in the pediatric post-anesthesia care unit for 2 hours before being transferred to the pediatric surgery in-patient ward. Pain assessments were conducted by attending nurses using the Face, Legs, Activity, Cry, Consolability (FLACC) score at 10, 20, and 30 minutes, and at 1, 2, and 6 hours postoperatively.^15^ Analgesia was not routinely administered to all patients. Instead, if a patient's FLACC score was ≥ 4, indicating inadequate analgesia, 1 mg/kg intravenous tramadol was administered as the first-line rescue analgesic.^14^^,^^16^ If the pain score remained ≥ 4 following tramadol administration, 15 mg/kg intravenous paracetamol was given as a second-line intervention.

Prior to discharge, parents were educated on the use of the Wong-Baker Pain Scale and provided with a printed copy of the scale to use at home.^15^ They were instructed to assess their child’s pain and, if the Wong-Baker score was 4 or higher, to administer 10 mg/kg oral ibuprofen.

Follow-up phone calls were conducted at 16 and 24 hours postoperatively to inquire about Wong-Baker pain scores, any use of analgesics, and overall patient comfort. During the 24-hour follow-up, parental satisfaction regarding postoperative pain management was recorded using a 3-point scale: not satisfied (1), partially satisfied (2), and very satisfied (3).

Patients were monitored for any complications related to the QLB both during their hospital stay and throughout the 24-hour postoperative follow-up period, and any adverse events were recorded.

### Sample size calculation

A pilot study involving five patients per group was conducted to estimate the effect size using the Confidence Interval (CI) approach described by Cocks et al.^17^ The sample size was calculated using the G*Power program, version 3.1 (Heinrich-Heine University, Duesseldorf, Germany), for a two-way repeated measures within-between interaction multivariate analysis of variance test, with α = 0.05, and power (1-β) = 0.80. The outcomes considered for sample size estimation were the FLACC score, measured at six postoperative time points, and the Wong-Baker score, measured at two time points. The effect size f(V) was determined to be 0.44 based on the pilot study. A sample size of 36 per group was calculated, and accounting for a 20% loss to follow-up, the total number of participants required was determined as 86 patients.

### Statistical analysis

Categorical variables were presented as frequencies and percentages. Continuous variables were presented as mean (SD) or median (Interquartile Range [IQR]). Normality of distribution was assessed using both visual methods (histograms, Q-Q plots) and analytical methods (Shapiro-Wilk test). Independent samples *t*-test or Mann-Whitney *U* test was utilized to compare continuous variables. Chi-Squared and Fisher's Exact test were employed to analyze categorical variables, as appropriate. Odds ratios were derived from contingency tables.

A non-parametric rank-based analysis of variance test type statistic for factorial longitudinal data was used to assess the interaction effect between time points and scores within the two groups with “nparLD” package version 2.2.^18^^,^^19^ Relative Treatment Effects (RTE) were calculated for each group at each time point. An RTE reflects the probability that a randomly chosen score from that group and time point is higher than a randomly chosen score from the entire sample. An RTE of 0.5 indicates no deviation from the overall average. For pain scores, RTE < 0.5 indicates lower scores, and RTE > 0.5 indicates higher scores.

Effect sizes were also calculated using Cliff’s Delta, which quantifies the probability that a randomly selected observation from one group is higher than one from the other group. Effect sizes were interpreted as negligible (< 0.15), small (0.15–0.33), medium (0.33–0.47), or large (> 0.47). Due to the non-normal distribution of the data, Cliff’s Delta was chosen over Cohen’s *d* as a more robust measure for non-parametric data.^20^

Post hoc pairwise comparisons were performed using the Wilcoxon signed-rank test, with Bonferroni correction applied to adjust for multiple comparisons among all condition pairs where significant overall differences were observed. These analyses were exploratory and aimed at identifying specific group differences. Additionally, postoperative time without analgesics and time to first analgesic requirement (any rescue analgesia) were compared between the two groups using Kaplan-Meier analysis and the log-rank test. All statistical analyses were performed using *R* Statistical Software, version 4.3.1 (R Foundation for Statistical Computing, Vienna, Austria) with the packages “survival”, “nparLD”, “ggplot2”, and “effsize”. The p-value of < 0.05 indicated statistical significance and all p-values were two-sided.

## Results

**Figure 2:**
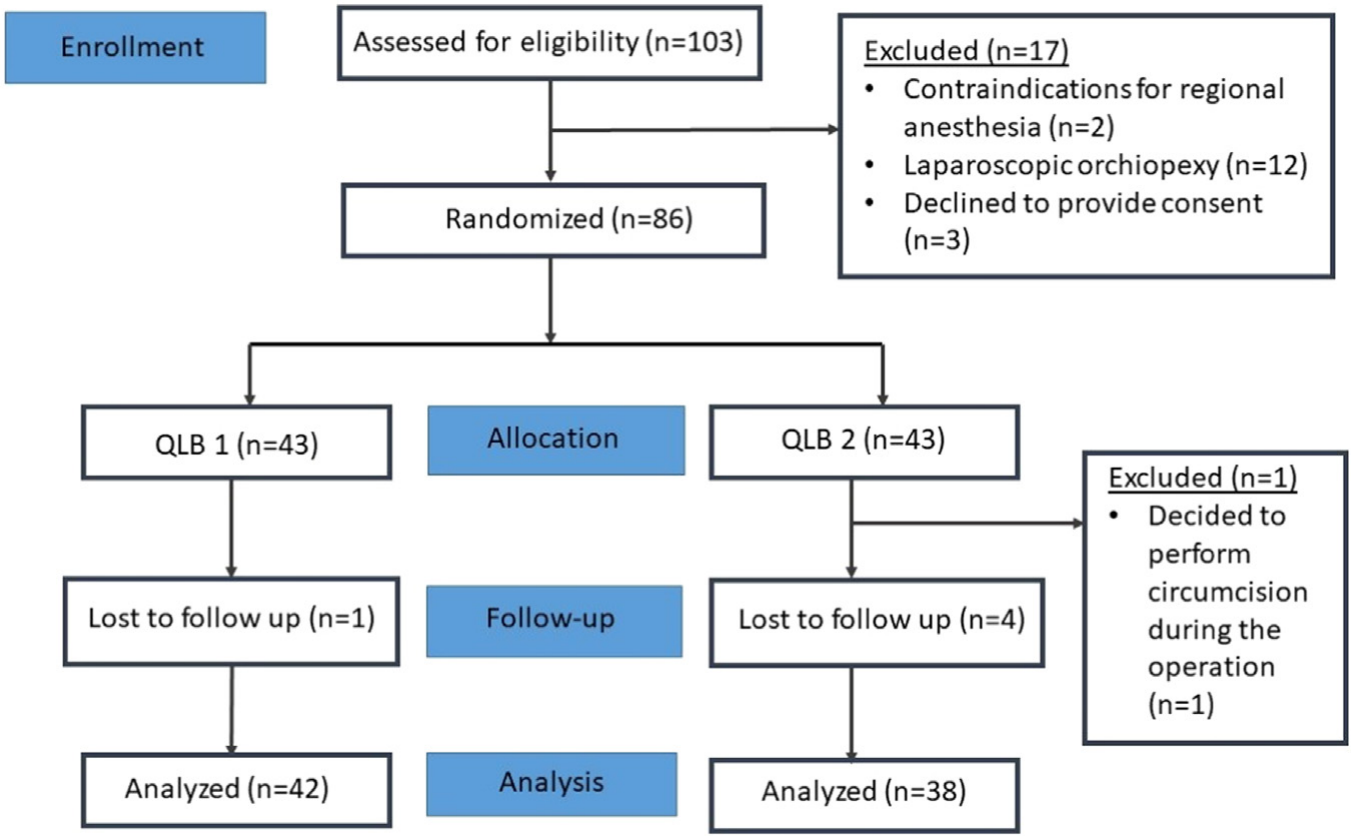
CONSORT diagram.

Figure 2 demonstrates the CONSORT diagram for the enrolment process of the study. Analyses were conducted on 80 patients, with 42 allocated to the lateral group (QLB-1) and 38 to the posterior group (QLB-2).

Baseline characteristics, the time between block and incision, the duration of surgery, and intraoperative hemodynamic parameters were similar between the two groups (Table 1, Supplementary Table 1). No significant interaction effect on intraoperative hemodynamic parameters was observed between the two QLB techniques across time intervals (Supplementary Fig. 1 and 2). Regarding the intraoperative usage of remifentanil, no statistically significant differences were observed in both groups (Supplementary Table 2).

**Table 1:** Demographic and clinical data.

	QLB1 (n = 42)	QLB2 (n = 38)	p-value
**Median age (IQR) in months**	42 (22‒69)	48 (36‒82)	0.078^a^
**Median weight (IQR) in kilograms**	17 (12‒22)	19 (13‒24)	0.347^a^
**Mean height (SD) in centimeters**	101 ± 16	107 ± 18	0.130^b^
**Median body surface area (IQR) in m^2^**	0.70 (0.56‒0.83)	0.75 (0.55‒0.88)	0.312^a^
**Median start time between block and incision (IQR) in minutes**	15 (13‒18)	15 (12‒20)	0.794^a^
**Median surgery duration (IQR) in minutes**	94.5 (89.0‒100.5)	96.0 (92.5‒100.7)	0.130^a^

IQR, Interquartile Range; SD, Standard Deviation; QLB, Quadratus Lumborum Block.

a Mann-Whitney-*U* test.

b Independent Samples *t*-test.

**Figure 3:**
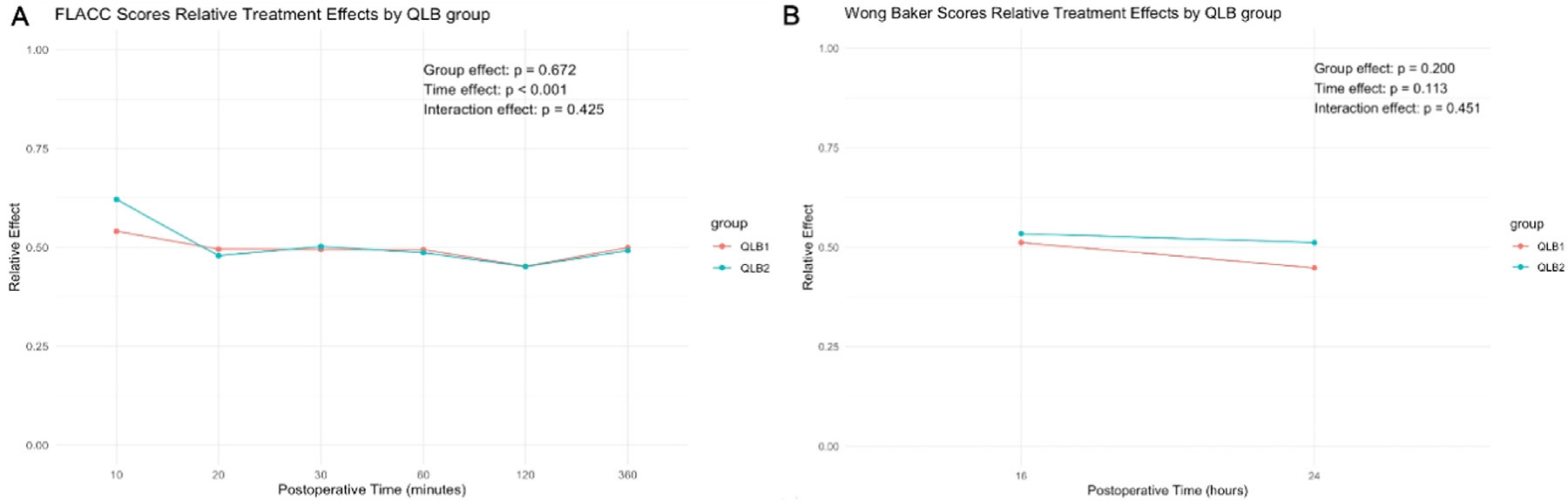
Changes in relative treatment effects of QLB groups over postoperative time for (A) FLACC, and (B) Wong-Baker scores.

FLACC and Wong-Baker scores were comparable between two groups at any recorded time interval (p > 0.05) (Table 2). Relative treatment effects were calculated for each group and the interaction effects between groups and postoperative time intervals measurements were not statistically significant (FLACC score p-interaction: 0.425, Wong-Baker score p-interaction: 0.451) (Fig. 3A‒3B; Supplementary Table 3). Clinically adequate analgesia, as indicated by total mean FLACC and Wong-Baker scores below four, was achieved in both study groups. Mean total FLACC ([QLB-1] 2.86 ± 4.69 vs. [QLB-2] 2.87 ± 3.71, p = 0.466; Cliff’s delta = -0.086, 95% CI: -0.313 to 0.150) and Wong-Baker ([QLB-1] 0.86 ± 2.03 vs. [QLB-2] 1.24 ± 1.85, p = 0.151; Cliff’s delta = -0.149, 95% CI: -0.345 to 0.060) scores were similar between two groups. Furthermore, parental satisfaction scores were similar between the groups (p = 0.400) (Table 2). In post-hoc pairwise comparisons, no statistically significant difference was observed at each time interval for either group (Supplementary Table 4 and 5). In the subgroup of patients older than 7 years, there were no significant differences in FLACC scores compared to patients younger than 7 years (Supplementary Table 6).

**Table 2:** Postoperative pain and parent satisfaction scores.

	QLB1 (n = 42)		QLB2 (n = 38)		p-value^a^	Cliff’s delta (95% CI)
	Mean (SD)	Median [IQR]	Mean (SD)	Median [IQR]		
**Total FLACC score**	2.86 (4.69)	1 [1‒5]	2.87 (3.71)	2 [1‒5]	0.466	−0.086 (−0.313, 0.150)
10^th^ min	1.38 (2.87)	0 [0‒0]	1.55 (2.29)	0 [0‒3]	0.214	−0.131 (−0.333, 0.082)
20^th^ min	0.52 (1.47)	0 [0‒0]	0.45 (1.43)	0 [0‒0]	0.657	0.033 (−0.115, 0.180)
30^th^ min	0.50 (1.44)	0 [0‒0]	0.55 (1.50)	0 [0‒0]	0.846	−0.016 (−0.175, 0.145)
60^th^ min	0.45 (1.21)	0 [0‒0]	0.32 (0.90)	0 [0‒0]	0.822	0.018 (−0.136, 0.171)
2^nd^ hour	0.36 (1.62)	0 [0‒0]	0.21 (1.14)	0 [0‒0]	0.949	−0.003 (−0.101, 0.095)
6^th^ hour	0.69 (1.81)	0 [0‒0]	0.55 (1.61)	0 [0‒0]	0.841	0.016 (−0.138, 0.169)
**Total Wong Baker score**	0.86 (2.03)	0 [0‒0]	1.24 (1.85)	0 [0‒3]	0.151	−0.149 (−0.345, 0.060)
16^th^ hour	0.67 (1.51)	0 [0‒0]	0.79 (1.60)	0 [0‒0]	0.642	−0.043 (−0.225, 0.141)
24^th^ hour	0.19 (0.74)	0 [0‒0]	0.45 (1.01)	0 [0‒0]	0.084	−0.135 (−0.286, 0.023)
**Parent satisfaction score**	2.90 (0.30)	3 [3‒3]	2.84 (0.37)	3 [3‒3]	0.400	0.063 (−0.087, 0.210)

Data are displayed as mean (SD), median [IQR], or n/total n (%).

CI, Confidence Interval; IQR, Interquartile Range; P, Percentile; SD, Standard Deviation; min, Minute.

a Mann-Whitney-*U* test.

**Figure 4:**
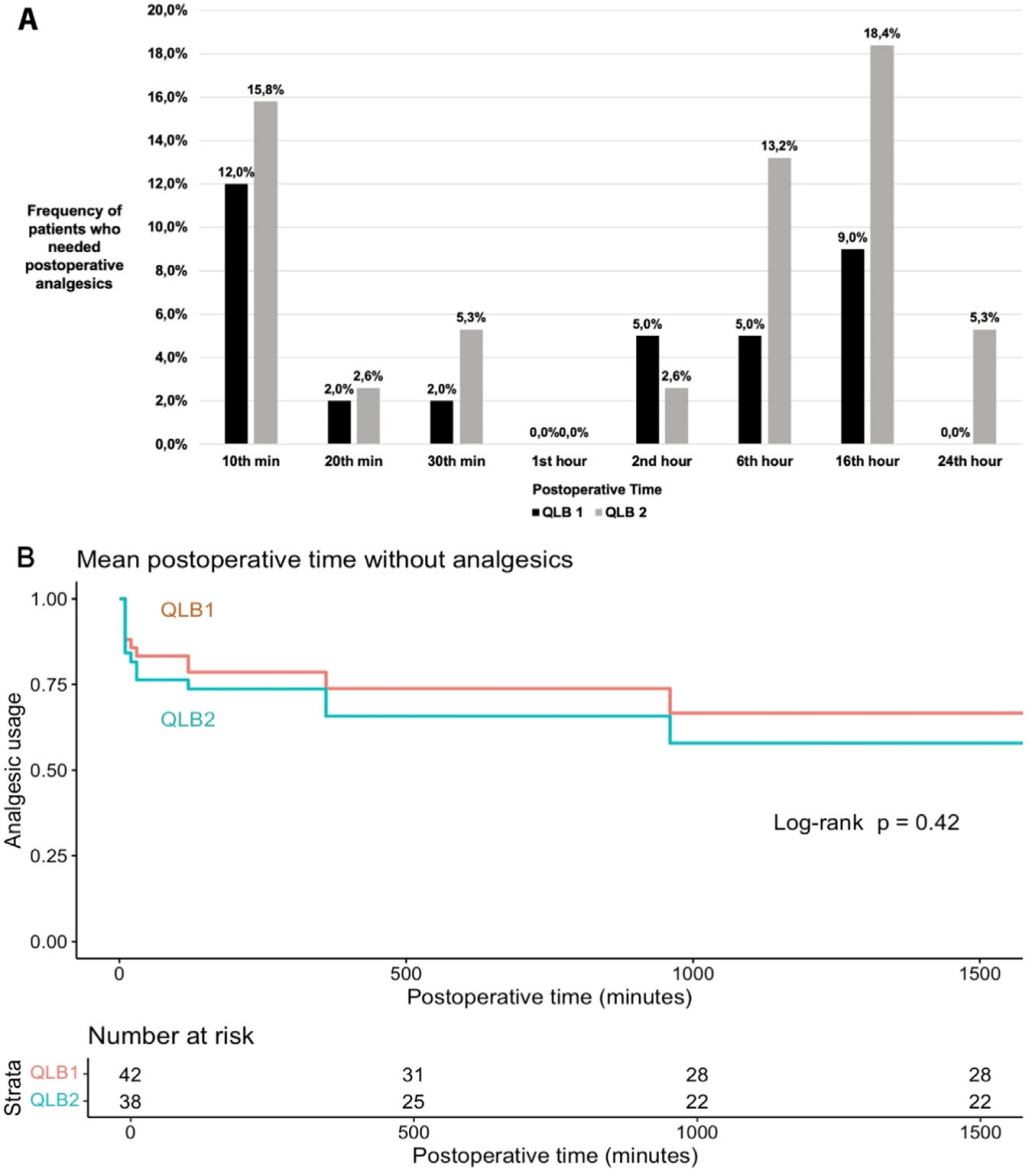
(A) Postoperative analgesic utility, (B) Kaplan-Meier plot for mean postoperative time without analgesics.

During postoperative follow-up, it was noted that 14 patients from the QLB-1 group and 16 patients from the QLB-2 group required additional analgesics (p = 0.563). No statistically significant differences were observed in the number of patients requiring postoperative analgesics between the two groups at any time in 24 hours (p > 0.05) (Fig. 4A, Supplementary Table 7). Moreover, the durations of analgesic-free interval were similar in both groups (p = 0.421) (Fig. 4B, Supplementary Table 8). No hemodynamic abnormalities, complications, or side effects were observed in either group throughout the perioperative period.

## Discussion

This study compared the clinical effectiveness of lateral and posterior QLB. The main findings of our study were as follows: there was no statistically significant difference in (1) Postoperative pain scores in 24 hours, (2) Perioperative analgesic requirements, and (3) Parental satisfaction between the two blocks, demonstrating comparable postoperative analgesia in pediatric patients undergoing open orchiopexy.

To our knowledge, this is the first double-blind, prospective, randomized study comparing perioperative analgesic efficacy of lateral and posterior QLB in children. QLB is an effective fascial plane block for lower abdominal surgeries and orchiopexy in pediatric patients.^2^^,^^4^, ^5^, ^6^ However, in the literature, QLB techniques vary across studies, highlighting the need for direct comparative evaluations of different QLB approaches.^2^^,^^9^

The lateral QLB is performed at the anterolateral border of the QLM, specifically at its junction with the transversalis fascia.^13^ The mechanism of action is thought to involve the spread of injectate into the Transversus Abdominis Plane (TAP) and potentially further through the anterior TLF into the paravertebral space.^7^^,^^8^^,^^11^ In contrast, the posterior QLB targets the posterior aspect of the QLM, aiming at the LIFT, and is proposed to spread via the middle TLF.^11^^,^^13^ In addition, the TLF, which has a high-density network of sympathetic fibers and mechanoreceptors, is considered to be a contributing factor in the effects of QLB.^7^^,^^8^

An imaging study with Computed Tomography (CT) demonstrated that, in both lateral and posterior QLB, the injected solution was consistently observed in the TAP and intercostal planes, particularly around the 10^th^ and 11^th^ ribs.^11^ These regions correspond to the pathways of the ilioinguinal, iliohypogastric, subcostal, and lower intercostal nerves.^11^ This observation aligns with the findings of anatomical studies and suggests a plausible mechanism of action for these blocks in patients undergoing abdominal surgery.^8^^,^^21^^,^^22^ We hypothesized that posterior QLB could provide better analgesia due to its potentially broader spread, as demonstrated by imaging studies using CT and contrast-enhanced MRI.^11^^,^^12^ However, these studies primarily focused on the anatomical distribution of the injectate rather than comparing clinical analgesic outcomes.^11^^,^^12^ In contrast to our expectations, our findings did not demonstrate a significant analgesic advantage of posterior over lateral QLB in pediatric patients undergoing orchiopexy.

Our previous study compared the posterior TAP block and lateral QLB, showed that lateral QLB is clinically more effective than the TAP block in children undergoing open orchiopexy.^5^ In the current study, the comparable efficacy observed between lateral and posterior QLB further reinforces the clinical value of QLB techniques overall and highlights their potential advantage over the TAP block for orchiopexy. In line with these findings, a recent meta-analysis demonstrated that QLB reduces postoperative pain scores and the need for rescue analgesia compared to caudal block and other peripheral nerve blocks, without increasing side effects after lower abdominal surgery in children.^2^ However, in the subgroup analysis, there was no consistent difference between the QLB techniques due to notable study heterogeneity.^2^

There are limited number of clinical studies comparing the analgesic efficacy of posterior and lateral QLB in adults, no data in children exists.^9^^,^^23^^,^^24^ Li et al. compared lateral and posterior QLB with a control group, including 32 patients in each group aged 18–70 years, undergoing laparoscopic renal surgery. Unlike our study, all patients received routine flurbiprofen and a basal sufentanil infusion (1.25 mcg/mL at 0.5 mL/h) via a Patient-Controlled Analgesia (PCA) pump. Both QLB approaches provided a decrease in somatic and visceral pain intensity for up to 24 hours after surgery compared to the control group; however, they did not lead to a reduction in total opioid consumption, which was attributed to the continuous PCA basal infusion administered across all groups. Furthermore, there was no statistically significant difference in analgesic efficacy between the lateral and posterior QLB techniques.^23^

In another study, lateral and posterior QLB approaches were compared in overall 57 patients aged 20‒60 undergoing laparoscopic cholecystectomy. Tenoxicam was routinely administered before the end of surgery. Postoperative analgesia was managed with intravenous PCA tramadol, and paracetamol was used as rescue medication. The blocks did not result in any significant differences in pain scores at any postoperative time point, nor in intraoperative or postoperative analgesic consumption.^24^ Our findings align with those reported in adult studies, showing that lateral and posterior QLB techniques yield comparable outcomes.^23^^,^^24^ Therefore, these results may extend across different age groups and surgical settings. However, direct comparison of effect sizes is limited by differences in patient demographics, surgical procedures, and analgesic protocols.

On the other hand, differences between pediatric and adult regional anesthesia should be considered. Pediatric regional anesthesia is generally more technically challenging than in adults, although ultrasound image interpretation and needle visualization tend to be easier. Specifically regarding QLB, the spread of LA may differ due to variations in muscle and fascial planes compared to adults.^25^ The recommended LA concentration and volume for pediatric QLB is 0.2–0.5 mL/kg of 0.25% bupivacaine, 0.25% levobupivacaine, or 0.2% ropivacaine in accordance with the current guideline.^16^ Additionally, only a limited correlation has been demonstrated between the postoperative pain scores and the volume of LA administered.^26^

In children undergoing orchiopexy, previous data indicated that the most intense period of postoperative pain occurs within the initial 24 hours following the procedure.^27^ Consequently, our primary objective centered on evaluating perioperative pain and analgesic usage within critical 24-hour timeframe. In our study, both approaches demonstrated clinically effective for analgesia, with no significant disparities observed in pain scores between the two groups. The fact that both blocks provided sufficient analgesia to minimize postoperative distress and reduce the overall need for additional analgesics highlights their practical value in pediatric patients, where undertreated pain is known to have lasting effects on pain perception and long-term outcomes.^28^

Nevertheless, it is worth noting that 14 patients in the lateral QLB group and 16 in the posterior QLB group required additional analgesic intervention, although there were no statistically significant distinctions in any time or duration of analgesic-free intervals within these two groups. We postulate that the primary reason for the demand for supplemental analgesics can be attributed to anatomical variations in the dispersion of LA within the TLF.^8^^,^^11^ Secondary factors may include disparities in the intensity of surgical stimuli and areas of uncovered innervation, which may arise due to the intricate innervation of the testis, spermatic duct, and scrotum.^3^^,^^29^

Innervation of the spermatic cord is supplied by three main sources: the superior spermatic nerves from the renal and intermesenteric plexus, the median spermatic nerves from the superior hypogastric plexus, and the inferior spermatic nerves from the pelvic plexus. Innervation of the testis and scrotum includes: (1) Somatic and sensory innervation through the iliohypogastric, ilioinguinal, genitofemoral, and pudendal nerves (from the L1–L2 and S2–S4 roots); (2) Parasympathetic innervation from the S2‒S4 segments; (3) Sympathetic innervation from the T10‒L1 roots, which embryologically share innervation with the kidney.^29^^,^^30^ However, both lateral and posterior QLB predominantly provide analgesia by involving dermatomes from T7 to L1.^7^^,^^26^^,^^31^ This may not be sufficient for scrotal incision due to the complex scrotal innervation from the genitofemoral, pudendal, posterior femoral cutaneous, and ilioinguinal nerves originating from L1‒S3.^29^

QLB is generally recognized as a reliable regional technique,^2^^,^^26^ and no complications or side effects were observed in our study. Nevertheless, it is crucial to be aware of potential complications. Hemodynamic side effects and motor block can occur due to the dispersion of LA into paravertebral spaces and the lumbar plexus.^2^^,^^9^ The risks of retroperitoneal hematoma and solid organ damage, such as liver, kidney, and intestine, should not be overlooked.^7^^,^^9^ LA toxicity should be considered, especially after the bilateral block performance.^2^^,^^8^ Lastly, postoperative nausea/vomiting and urinary retention can also manifest. A recent meta-analysis has shown comparable results in postoperative nausea/vomiting and urinary retention between QLB or non-QLB in children.^10^

The limitations of our study were as follows: (1) We could not assess the level of sensory block during intraoperative and postoperative periods. (2) Pain assessment and analgesic administration after the 6^th^ hour were determined by parents, as the orchiopexy procedure was performed on an outpatient basis. Using the Wong-Baker score for post-discharge pain evaluation was necessitated because the FLACC score was deemed unsuitable for parental assessment. (3) While the FLACC score is typically more appropriate for children under seven, we employed it across all age groups to maintain a consistent approach to pain assessment. The mean age of the patients included in our study was 51 months; therefore, we believe that using the FLACC score in older age groups did not significantly impact the study's results. Additionally, we performed a subgroup analysis comparing children older than seven years with those younger than seven, and the results indicated no significant differences in FLACC scores between the two groups. (4) The sample size for this study was initially determined based on a pilot study, aiming to assess both between-group differences and within-group changes. Final study calculations confirmed that sufficient statistical power was achieved for detecting between-group differences; however, the power to detect within-group changes may have been limited.

## Conclusion

This study demonstrated that both lateral and posterior QLB provide effective postoperative analgesia in pediatric patients undergoing orchiopexy, with no significant differences between them in terms of 24-hour postoperative pain scores, rescue analgesic requirements, parental satisfaction, or complications. Consequently, either technique may be considered based on the patient's clinical condition, without the need to reposition the patient after anesthesia induction. The lateral QLB may be preferred for orchiopexy due to its relative technical simplicity, whereas the posterior approach requires greater expertise. Nonetheless, it is noteworthy that there is limited research directly comparing these two techniques. Therefore, further studies are needed to better understand their comparative efficacy and safety profiles.

## Authors' contributions

Ozgecan P. Zanbak Mutlu made substantial contributions to the conception and design of the study, data collection, interpretation, and drafting of the manuscript. She also critically revised the work for important intellectual content and provided final approval of the version to be published. Pinar Kendigelen contributed significantly to the implementation of blocks. Ayse C. Tutuncu played a key role in the study's conception and design, implementation of blocks, and critical revision of the manuscript.

## Funding

This research did not receive any specific grant from funding agencies in the public, commercial, or not-for-profit sectors.

## Conflicts of interest

The authors declare no conflicts of interest.

## Appendix Supplementary materials

Image, application 1
