# Supplementary material for: Lateral versus posterior quadratus lumborum block in children undergoing open orchiopexy: a double-blind randomized clinical trial
Source: Braz J Anesthesiol. 2025 Jul 5;75(6):844661. doi: 10.1016/j.bjane.2025.844661 (PMC12332913; doi:10.1016/j.bjane.2025.844661)

**BJAN-D-25-00093_Supplementary Material**

**Supplementary Table 1** Intraoperative hemodynamic parameters.

|  | **QLB1 (n = 42)** | **Relative treatment effect** | **QLB2 (n = 38)** | **Relative treatment effect** | **p-value between groups** |
| --- | --- | --- | --- | --- | --- |
| **Mean arterial pressure (IQR)** |  |  |  |  |  |
| Pre-incision | 67.5 (63.0‒71.0) | 0.490 | 67.5 (63.0‒73.7) | 0.517 | 0.440^a^ |
| Post-incision |  |  |  |  |  |
| 5^th^ min | 72.1 (59.5‒77.2) | 0.565 | 70.5 (64.2‒77.0) | 0.593 | 0.965^a^ |
| 10^th^ min | 70.6 (66.7‒79.4) | 0.637 | 70.0 (65.0‒76.0) | 0.560 | 0.352^a^ |
| 20^th^ min | 69.5 (62.5‒75.3) | 0.548 | 67.5 (62.2‒74.7) | 0.540 | 0.739^a^ |
| 30^th^ min | 66.5 (60.5‒71.7) | 0.477 | 65.5 (60.0‒72.0) | 0.490 | 0.839^a^ |
| 45^th^ min | 63.5 (60.2‒68.0) | 0.404 | 63.0 (60.0‒68.0) | 0.425 | 0.908^a^ |
| 60^th^ min | 63.5 (59.0‒69.7) | 0.398 | 63.5 (60.2‒68.0) | 0.420 | 0.927^a^ |
| p-value |  | <0.001^b^ |  | <0.001^b^ | 0.915^c^ |
| **Heart rate (IQR)** |  |  |  |  |  |
| Pre-incision | 102.0 (94.0‒116.0) | 0.535 | 100.0 (94.5‒116.0) | 0.506 | 0.900^a^ |
| Post-incision |  |  |  |  |  |
| 5^th^ min | 107.0 (93.0‒126.0) | 0.579 | 106.5 (93.5‒121.5) | 0.565 | 0.650^a^ |
| 10^th^ min | 108.5 (93.2‒120.0) | 0.581 | 107.5 (90.5‒118.7) | 0.558 | 0.881^a^ |
| 20^th^ min | 106.5 (92.7‒113.5) | 0.530 | 107.5 (90.5‒118.7) | 0.522 | 0.769^a^ |
| 30^th^ min | 101.5 (94.0‒113.5) | 0.466 | 100.5 (96.5‒110.0) | 0.496 | 0.946^a^ |
| 45^th^ min | 100.0 (91.0‒106.7) | 0.398 | 100.0 (92.0‒104.0) | 0.411 | 0.758^a^ |
| 60^th^ min | 99.5 (89.0‒107.0) | 0.392 | 97.5 (89.2‒105.7) | 0.392 | 0.725^a^ |
| p-value |  | <0.001^b^ |  | <0.001^b^ | 0.810^c^ |

IQR, Interquartile Range; min, Minute; QLB, Quadratus Lumborum Block; RTE, Relative Treatment Effect.

^a^ Mann-Whitney-*U* test.

^b^ Robust ANOVA type statistics for time effect.

^c^ Modified ANOVA type statistic for the whole-plot factors.

**Supplementary Table 2** Intraoperative usage of remifentanil.

|  | **QLB1 (n = 42)** | **QLB2 (n = 38)** | **OR (95% CI)^a^** | **p-value** |
| --- | --- | --- | --- | --- |
| **Number of patients taking remifentanil, n (%)** |  |  |  |  |
| Total | 9 (21.4) | 12 (31.6) | 1.69 (0.62‒4.63) | 0.303^b^ |
| Pre-incision | 0 (0.0) | 0 (0.0) | NA | NA |
| 5^th^ min | 3 (7.1) | 5 (13.2) | 1.97 (0.44‒8.87) | 0.467^c^ |
| 10^th^ min | 5 (11.9) | 7 (18.4) | 1.67 (0.48‒5.79) | 0.415^b^ |
| 20^th^ min | 7 (16.7) | 7 (18.4) | 1.13 (0.36‒3.58) | 0.837^b^ |
| 30^th^ min | 6 (14.3) | 6 (15.8) | 1.12 (0.33‒3.84) | 0.851^b^ |
| 45^th^ min | 5 (11.9) | 5 (13.2) | 1.12 (0.30‒4.22) | 0.866^b^ |
| 60^th^ min | 2 (4.8) | 5 (13.2) | 3.03 (0.55‒16.6) | 0.248^c^ |
| **Median total remifentanil dose (IQR), (mcg/kg)** | 3.00 (1.75‒6.5) | 4.00 (2.88‒5.38) |  | 0.592^d^ |

OR, Odds Ratio; CI, Confidence Interval; min, Minute

^a^ Odds ratios were given according to contingency tables.

^b^ Chi-Squared test

^c^ Fisher's Exact test

^d^ Mann-Whitney *U* test

**Supplementary Table 3** Relative treatment effects.

|  | **QLB1 (n = 42)** | **QLB2 (n = 38)** | **p-interaction^a^** |
| --- | --- | --- | --- |
| **FLACC score** | Relative treatment effect | Relative treatment effect | 0.425^a^ |
| 10^th^ min | 0.541 | 0.621 |  |
| 20^th^ min | 0.495 | 0.478 |  |
| 30^th^ min | 0.494 | 0.502 |  |
| 60^th^ min | 0.494 | 0.486 |  |
| 2^nd^ hour | 0.452 | 0.451 |  |
| 6^th^ hour | 0.499 | 0.491 |  |
| **Wong-Baker Score** |  |  | 0.451^a^ |
| 16^th^ hour | 0.511 | 0.534 |  |
| 24^th^ hour | 0.448 | 0.511 |  |

QLB, Quadratus Lumborum Block.

^a^ Robust ANOVA-type statistic for group and time interaction.

**Supplementary Table 4** Posthoc pairwise comparisons of FLACC scores of the QLB1 group.

| **Pairwise comparisons^a^** | **Test statistic** | **p-value^b^** |
| --- | --- | --- |
| **FLACC score** |  |  |
| 10^th^ min – 20^th^ min | 55.0 | 1.000 |
| 10^th^ min – 30^th^ min | 53.0 | 0.828 |
| 10^th^ min – 60^th^ min | 76.0 | 1.000 |
| 10^th^ min – 2^nd^ hour | 50.0 | 1.000 |
| 10^th^ min – 6^th^ hour | 23.5 | 1.000 |
| 20^th^ min – 30^th^ min | 14.5 | 1.000 |
| 20^th^ min – 60^th^ min | 36.5 | 1.000 |
| 20^th^ min – 2^nd^ hour | 21.5 | 1.000 |
| 20^th^ min – 6^th^ hour | 45.5 | 1.000 |
| 30^th^ min – 60^th^ min | 24.0 | 0.534 |
| 30^th^ min – 2^nd^ hour | 21.5 | 1.000 |
| 30^th^ min – 6^th^ hour | 39.5 | 1.000 |
| 60^th^ min – 2^nd^ hour | 15.5 | 1.000 |
| 60^th^ min – 6^th^ hour | 41.5 | 1.000 |
| 2^nd^ hour – 6^th^ hour | 21.5 | 1.000 |

^a^ Wilcoxon signed-rank test.

^b^ Adjusted with Bonferroni correction.

**Supplementary Table 5** Posthoc pairwise comparisons of FLACC scores of the QLB2 group.

| **Pairwise comparisons^a^** | **Test statistic** | **p-value^b^** |
| --- | --- | --- |
| **FLACC score** |  |  |
| 10^th^ min – 20^th^ min | 105.0 | 0.166 |
| 10^th^ min – 30^th^ min | 99.0 | 0.418 |
| 10^th^ min – 60^th^ min | 134.5 | 0.090 |
| 10^th^ min – 2^nd^ hour | 121.0 | 0.096 |
| 10^th^ min – 6^th^ hour | 28.5 | 0.351 |
| 20^th^ min – 30^th^ min | 8.5 | 1.000 |
| 20^th^ min – 60^th^ min | 12.5 | 1.000 |
| 20^th^ min – 2^nd^ hour | 10.5 | 1.000 |
| 20^th^ min – 6^th^ hour | 21.5 | 1.000 |
| 30^th^ min – 60^th^ min | 19.0 | 1.000 |
| 30^th^ min – 2^nd^ hour | 15.5 | 1.000 |
| 30^th^ min – 6^th^ hour | 28.5 | 1.000 |
| 60^th^ min – 2^nd^ hour | 6.0 | 1.000 |
| 60^th^ min – 6^th^ hour | 29.0 | 1.000 |
| 2^nd^ hour – 6^th^ hour | 20.0 | 1.000 |

^a^ Wilcoxon signed-rank test.

^b^ Adjusted with Bonferroni correction.

**Supplementary Table 6** FLACC score comparison by age.

|  | **Patients < 7-years (n = 62)** | | **Patients ≥ 7-years (n = 18)** | | **p-value**^a^ |
| --- | --- | --- | --- | --- | --- |
|  | **Mean (SD)** | **Median [IQR]** | **Mean (SD)** | **Median [IQR]** |  |
| **Total FLACC score** | 3.00 (4.52) | 0 [0‒5] | 2.39 (3.07) | 2 [0‒3] | 0.786 |
| 10^th^ min | 1.69 (2.81) | 0 [0‒3] | 0.67 (1.41) | 0 [0‒3] | 0.258 |
| 20^th^ min | 0.44 (1.34) | 0 [0‒0] | 0.67 (1.78) | 0 [0‒0] | 0.561 |
| 30^th^ min | 0.50 (1.46) | 0 [0‒0] | 0.61 (1.50) | 0 [0‒0] | 0.788 |
| 60^th^ min | 0.37 (1.13) | 0 [0‒0] | 0.44 (0.86) | 0 [0‒0] | 0.308 |
| 2^nd^ hour | 0.15 (1.02) | 0 [0‒0] | 0.78 (2.26) | 0 [0‒0] | 0.179 |
| 6^th^ hour | 0.81 (1.91) | 0 [0‒0] | 0.00 (0.00) | 0 [0‒0] | 0.057 |

Data are displayed as mean (SD), median [IQR], or n/total n (%).

CI, Confidence Interval; IQR, Interquartile Range; SD, Standard Deviation; min, Minute.

^a^ Mann-Whitney-*U* test.

**Supplementary Table 7** Postoperative analgesic utility.

|  | **QLB1**  **(n = 42)** | **QLB2**  **(n = 38)** | **p-value** |
| --- | --- | --- | --- |
| **Total number of patients who needed postoperative analgesics, n (%)** | 14 (33.3) | 16 (42.1) | 0.563^a^ |
| **Number of patients who needed postoperative analgesics, n (%)** |  |  |  |
| 10^th^ min | 5 (12) | 6 (15.8) | 0.858^a^ |
| 20^th^ min | 1 (2) | 1 (2.6) | 1.000^b^ |
| 30^th^ min | 1 (2) | 2 (5.3) | 0.602^b^ |
| 1^st^ hour | 0 (0) | 0 (0) | NA |
| 2^nd^ hour | 2 (5) | 1 (2.6) | 1.000^b^ |
| 6^th^ hour | 2 (5) | 5 (13.2) | 0.248^b^ |
| 16^th^ hour | 4 (9) | 7 (18.4) | 0.407^a^ |
| 24^th^ hour | 0 (0) | 2 (5.3) | 0.222^b^ |

min, Minute.

^a^ Chi-Squared test.

^b^ Fisher’s Exact test.

**Supplementary Table 8** Mean postoperative time without analgesics according to Kaplan-Meier analysis.

| **Block** | **Mean postoperative time without analgesics** | **p-value** |
| --- | --- | --- |
|  | **Minutes** |  |
| QLB1 | 1427.1 ± 848.4 | 0.421 |
| QLB2 | 1268.9 ± 900.2 |  |

QLB, Quadratus Lumborum Block.

**Supplementary Figure 1** Relative treatment effects of mean arterial pressure.


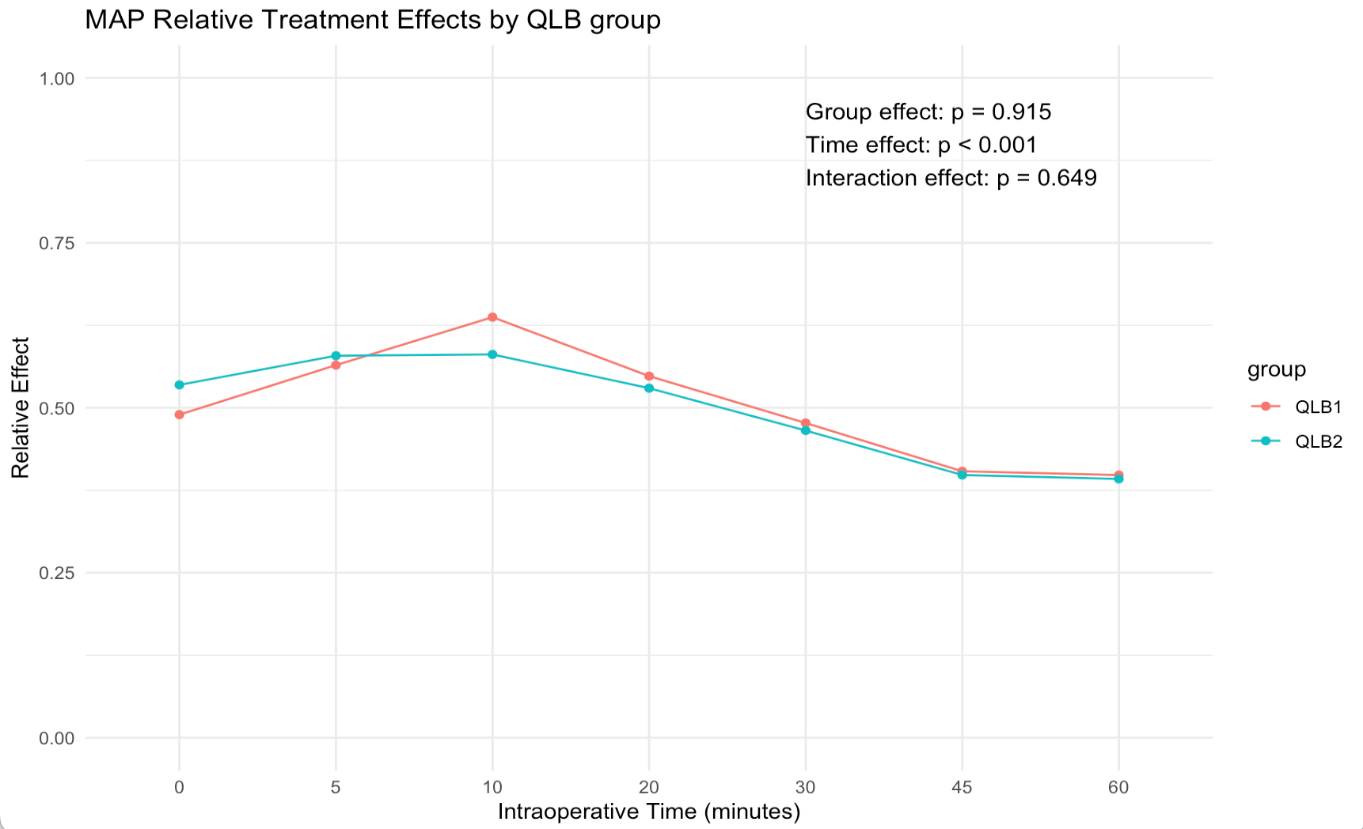


**Supplementary Figure 2** Relative treatment effects of heart rate.


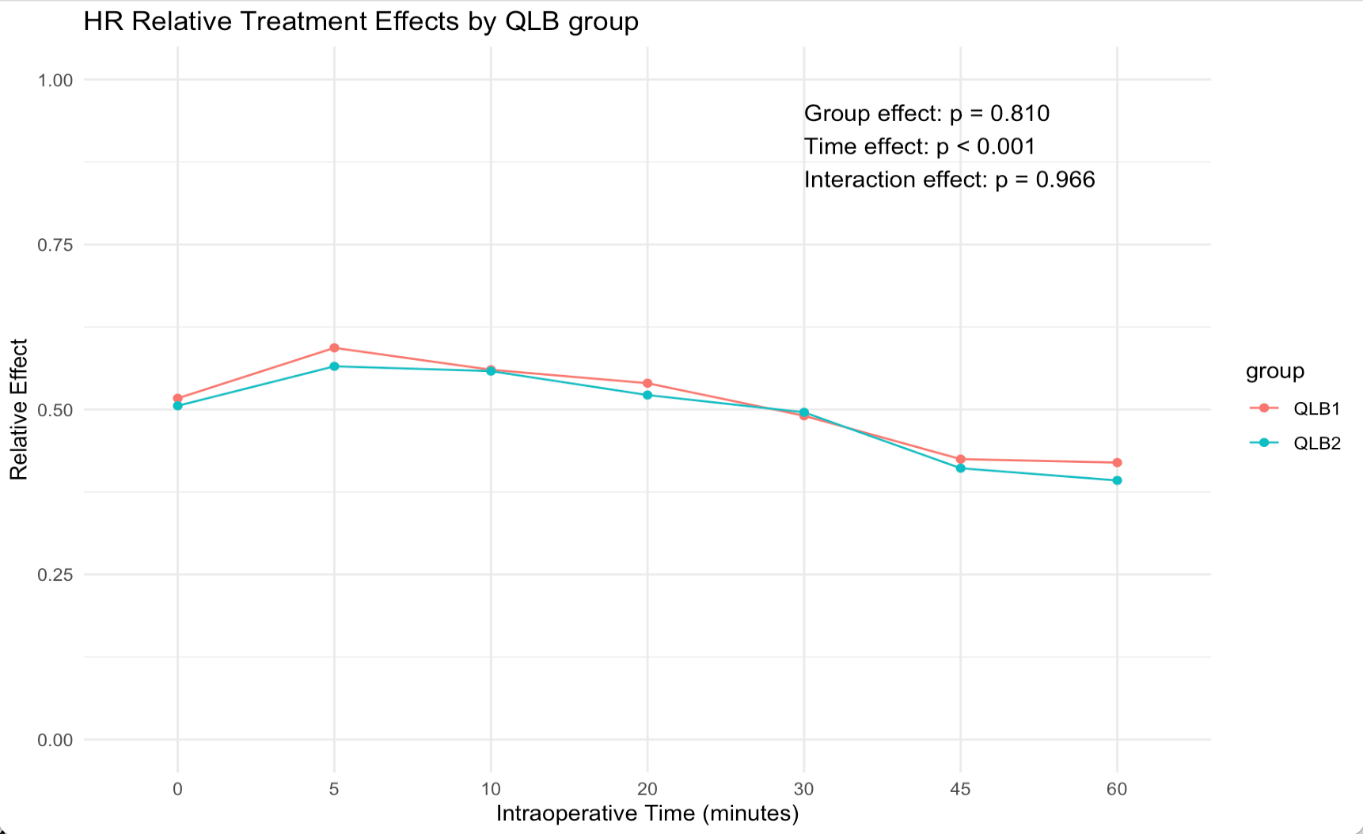

Supplement: Supplementary file 1 [file mmc1.docx]
